# Peculiarities of Zika Immunity and Vaccine Development: Lessons from Dengue and the Contribution from Controlled Human Infection Model

**DOI:** 10.3390/pathogens11030294

**Published:** 2022-02-25

**Authors:** Helton C. Santiago, Tertuliano A. Pereira-Neto, Marcela H. Gonçalves-Pereira, Ana C. B. Terzian, Anna P. Durbin

**Affiliations:** 1 Department of Biochemistry and Immunology, Institute of Biological Sciences, Federal University of Minas Gerais, Belo Horizonte 30270-901, MG, Brazil; tertuliano1@gmail.com (T.A.P.-N.); marcelahgpo@gmail.com (M.H.G.-P.); 2 Laboratory of Cellular Immunology, Rene Rachou Institute, Fiocruz, Belo Horizonte 30190-002, MG, Brazil; ana.terzian@fiocruz.br; 3 Johns Hopkins Bloomberg School of Public Health, Baltimore, MD 21205, USA; adurbin1@jhu.edu

**Keywords:** Zika, dengue, cross-reactivity, vaccine, pathogenesis, CHIM

## Abstract

The Zika virus (ZIKV) was first isolated from a rhesus macaque in the Zika forest of Uganda in 1947. Isolated cases were reported until 2007, when the first major outbreaks of Zika infection were reported from the Island of Yap in Micronesia and from French Polynesia in 2013. In 2015, ZIKV started to circulate in Latin America, and in 2016, ZIKV was considered by WHO to be a Public Health Emergency of International Concern due to cases of Congenital Zika Syndrome (CZS), a ZIKV-associated complication never observed before. After a peak of cases in 2016, the infection incidence dropped dramatically but still causes concern because of the associated microcephaly cases, especially in regions where the dengue virus (DENV) is endemic and co-circulates with ZIKV. A vaccine could be an important tool to mitigate CZS in endemic countries. However, the immunological relationship between ZIKV and other flaviviruses, especially DENV, and the low numbers of ZIKV infections are potential challenges for developing and testing a vaccine against ZIKV. Here, we discuss ZIKV vaccine development with the perspective of the immunological concerns implicated by DENV-ZIKV cross-reactivity and the use of a controlled human infection model (CHIM) as a tool to accelerate vaccine development.

## 1. Introduction

Zika virus (ZIKV) was initially isolated from a rhesus macaque in the Zika forest of Uganda in 1947 [1]. It is an arbovirus from the *Flaviviridae* family and, together with other Flaviviruses like dengue (DENV) and yellow fever (YFV), poses as one of the major public health problems in Latin America. Other important flaviviruses of public health concerns include West Nile virus (WNV) and Japanese encephalitis virus (JEV), which together with DENV and ZIKV, are considered emerging tropical viruses. ZIKV is transmitted by the female *Aedes aegypti* or *Aedes Albopictus* mosquito, both being widely distributed in Latin America [2]. However, other routes of transmission are also described including blood transfusion, sexual transmission, and transmission via breast milk [3,4,5].

Sporadic reports of natural Zika and/or serologic evidence of ZIKV infection have been reported since its discovery [6]. The first major outbreak of ZIKV was reported from the Island of Yap in Micronesia in 2007 where it was estimated that 72.6% of the population ≥3 years of age was infected, demonstrating the rapid transmission of ZIKV in a naïve population [7]. A second major outbreak occurred in French Polynesia from October of 2013 through early 2014, when it was estimated that 28,000 ZIKV infections occurred (~11% of the population) [8].

ZIKV began to circulate in Latin America between 2013 and 2014. In March of 2014, Chilean public health authorities confirmed that ZIKV infection was detected in cases reported in February, concurrent with the circulation of the virus in French Polynesia [9]. Indeed, the strain of ZIKV circulating in Latin America from 2014 to 2016 is related to the French Polynesia strain, which is estimated to have arrived in Latin America in 2013 [10]. Clinical cases of ZIKV started to be reported in Brazil in October 2014 after cases of disease presenting with low-grade fever, exanthema, pruritus, arthralgia, and limb edema tested negative for dengue, yellow fever, measles, rubella, enterovirus and chikungunya in Rio Grande do Norte state. After cases were also reported in Bahia state, the identification of ZIKV as the aetiological agent of the new disease was confirmed in May of 2015 [11]. In response, PAHO issued an epidemiological alert of ZIKV infection with recommendations for clinical management and prevention and control measures. The Brazilian Ministry of Health started to receive notification of increased frequencies of microcephaly in areas where ZIKV was circulating and an epidemiological investigation was started [12]. In December of 2015, PAHO, together with the Brazilian Ministry of Health, recognized the epidemiological association between ZIKV infection in pregnant women and microcephaly in newborns and released another epidemiological alert [13]. After confirmation of ZIKV-induced microcephaly, the WHO issued a Public Health Emergency of International Concern (PHEIC) on 1 February 2016, attracting greater attention and scientific resources for this epidemic. Anecdotal evidence of ZIKV and microcephaly began appearing in the literature in January of 2016 [14], and was confirmed by subsequent stronger epidemiological and virological evidence [15,16].

Several seroprevalence studies have shown that Zika incidence may have reached up to 70–80% of the population in Latin American countries and that its introduction was silent, especially when introduced in dengue-endemic regions. Recently, a new immunological survey of undergraduate students using humoral and cellular tests has identified ZIKV-positivity in more than 80% of samples that could at least partially differentiate DENV and ZIKV infections [17]. These numbers are not far from those reported in Brazil, where a serological survey estimated that ZIKV seroprevalence exceeded 60% in Salvador (state of Bahia) [18]. Likewise, the prevalence of flavivirus infections was estimated to be around 92% in the state of Ceará in 2018, of which only 37% were considered to be associated with DENV [19].

ZIKV has been reported in 87 countries with autochthonous transmission of the virus in the Americas, the Caribbean, Asia and Africa [20]. However, since the Latin American peak of infections in 2016, the number of reported cases has decreased dramatically. While in 2016, Brazil alone reported over 273,000 Zika cases to PAHO, in 2017 the number of cases dropped to 31,000. Since, it has varied from 18,000 to 31,000 cases per year, showing signs of stabilization [21]. Unfortunately, this means that ZIKV is still circulating and is probably under-reported. Nevertheless, Zika is still causing the devastating effects of Congenital Zika Syndrome (CZS). A serological survey in Recife (Pernambuco, Brazil), the epicenter of CZS, performed in 2018, estimated that while the prevalence of anti-ZIKV IgM in pregnant women in the general population was around 1.6%, therefore higher than expected for official case numbers, ZIKV-associated complicated pregnancies was around 7% [22], suggesting that health policies to address congenital Zika are still very relevant.

## 2. Clinical Presentation

The clinical presentation of ZIKV infection is mostly asymptomatic or oligosymptomatic. It is estimated that 80% of the cases do not seek health care [23]. In symptomatic cases, ZIKV generally causes a mild infection characterized by rash, low-grade fever, non-purulent conjunctivitis and myalgia [7,16]. Nearly all symptomatic patients from the Yap Island outbreak, for example, presented with rash (90%), arthritis/arthralgia (65%), and fever (65%). Unlike dengue or yellow fever, Zika does not cause hemorrhagic manifestations, vascular leak syndrome, or liver function abnormalities. Only approximately 19% of subjects found to be seropositive to ZIKV in a serosurvey from Yap Island recounted being symptomatic with a Zika-like illness [7]. In October of 2013, the largest outbreak of ZIKV recorded up to that time began in French Polynesia [8,24]. It was estimated that 28,000 ZIKV symptomatic infections occurred (~11% of the island population) with most infections presenting with low-grade fever, rash, arthralgia and conjunctivitis [24]. However, household surveys and serological investigations estimated that 73% of French Polynesia residents have been infected [7].

The French Polynesia outbreak revealed that ZIKV infection was also associated with Guillain–Barré Syndrome (GBS) with an estimated incidence of 1 up to 2.4 GBS cases per 10,000 ZIKV infections [8,24,25,26]. Similar numbers were estimated in the Latin American outbreak, which found 2.0 GBS cases per 10,000 ZIKV infections. It was calculated that ZIKV infection is associated with up to 10× higher incidence than those observed in the general population [27]. ZIKV-associated GBS was related to increased frequencies of facial weakness and paresthesia, dysphagia, shortness of breath, admission to intensive care unit and required mechanical ventilation when compared to non-ZIKV GBS [28].

The most devasting effect of Zika, however, is CZS. It was first noticed as an increased incidence of microcephaly in newborns from the Brazilian states where ZIKV was circulating [29]. The historical prevalence of microcephaly was around 0.6 cases per 10,000 live births and, in 2015, reached 2.8 cases per 10,000 [29]. Soon, it became clear that in addition to decreased head circumference and decreased birth weight, newborns from mothers who had gestational Zika developed several neurological, osteoskeletal, ophthalmic and many other abnormalities, including fetal death [30].

## 3. Immune Responses

**Figure 1:**
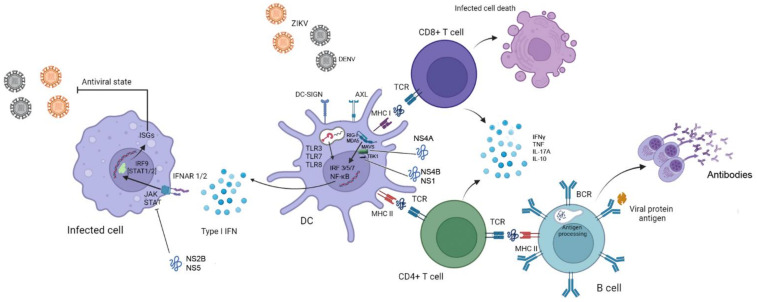
Immune response to DENV and ZIKV. ZIKV attaches to the target cell using DC-SIGN Axl receptors, which are used to mediate membrane fusion. Viral RNA is detected by PRRs, such as RIG-I and MDA-5 in the cytoplasm, and endosomal TLRs. The viral sensing mechanism induces the activation of the transcription factors NF-κB and IRFs, which mediate the production and secretion of interferons. Binding of type I IFNs to receptors (IFNAR1/2), especially in a bystander cell, initiates signaling cascades via JAK/STAT and the formation of a STAT/IRF9 trimer, which culminates in the production of multiple ISGs and induction of the cellular anti-viral state. Adaptive immunity is initiated after recognition of viral antigens presented via MHC class I or II, by CD8+ and CD4+ T cells, respectively, that produce cytokines to promote inflammation and exert other effector mechanisms, like killing of infected cells by CD8+ T cells. CD4+ T cells can also promote better antibody responses inducing class switch (inducing the production of IgG), affinity maturation and the differentiation of B cells into plasma cells. In contrast, non-structural proteins of the virus inhibit I IFN response by binding to MAVS (NS4a), inhibiting of TBK1 (NS1 and NS4b) or JAK (NS2b), and promoting the degradation of STAT2 (NS5). Abbreviations: PRRs—pattern recognition receptors, TLRs—Toll-like receptors, IRFs—interferon-related factors, ISGs—IFN-stimulated genes. Images from Servier Medical Art, licensed under a Creative Common Attribution 3.0 Generic License (http://smart.servier.com/; accessed on 10 Februrary 2022).

The interaction between the cells and the viruses occurs through pattern recognition receptors (PRRs), which recognize conserved and shared structures by pathogens, called pathogen-associated molecular patterns (PAMPs), which include lipids, proteins, carbohydrates, and nucleic acids [31]. In flavivirus infection, especially ZIKV, the most prominent PRRs involved in innate immunity activation are the cytoplasmic RLRs, RIG-I and MDA5, and endosomal TLRs such as TLR3, TLR7 and TLR8, involved in sensing ZIKV RNA [31,32,33,34,35,36]. These pathways activate the production of type I IFNs in viral infections, important mediators of a variety of effector mechanisms that contribute to the antiviral response (Figure 1). For example, mice which are deficient in IFNa receptor 1 (IFNAR1) are highly susceptible to ZIKV infection [37]. Lazear et al. showed that IFNaR1 was a key factor in resistance against ZIKV infection, but dependent on the downstream interferon-related factors (IRF) 3, 5 and 7. Although IRF3, 5 and 7 seem to be redundant in IFNa transduction, i.e., the depletion of each of these transduction factors individually did not impact mouse survival following ZIKV infection, the absence of the three transduction factors caused animals to succumb with high virus loads in the brain [37], showing that type I IFNs signaling is essential for immunity against ZIKV.

Type I IFN activates its receptor, triggering the activation of JAK1 and phosphorylation of STAT2. Two STAT2 molecules form a trimer with one IRF9 and migrate to the nucleus to identify and activate the production of the IFN-stimulated genes (ISGs), which induce the anti-viral state (Figure 1). However, ZIKV, as well as other flaviviruses, possess several mechanisms to evade the type I IFN pathway. For example, ZIKV NS4a binds to MAVS [38], an adaptive molecule that interacts with RIG-I to promote activation of TBK1 and downstream anti-viral signaling [39], inhibiting the production of type I IFN. The viral NS1 and NS4b proteins can also inhibit type I IFN production by inhibiting the phosphorylation of TBK1, a molecule downstream of the RIG-I-MAVS complex [40]. In addition, several mechanisms may also inhibit the type I and III IFN downstream signaling which are highly dependent on JAK-STAT signaling and IRF activation. The virus can inhibit JAK activation by NS2B [40], while NS5 possesses the ability to degrade STAT2 [41,42], impairing the expression of the interferon-stimulated genes (ISGs). Indeed, animals deficient in IFNAR1 downstream signaling, like animals deficient in STAT2 display important virus proliferation [43,44] (Figure 1).

The humoral and cellular adaptive immunity has also been shown to be activated during ZIKV infection. Adaptive immunity against ZIKV is very similar to that of DENV infection, including the ability of pathogen-specific antibodies to neutralize ZIKV. For flaviviruses, neutralizing antibodies target the envelope (E) protein, which has approximately 55% amino acid identity between ZIKV and DENV [45]. Neutralizing antibodies prevent virus attachment to the host cell and/or membrane fusion after virus uptake by endocytosis [46,47]. Neutralizing antibodies are considered the main mechanism of host resistance to flavivirus infections; however, antibodies have also been implicated in pathogenesis. For example, while high levels of neutralizing antibodies are able to confer protection against a specific virus, non-neutralizing antibodies may cause antibody-dependent enhancement (ADE) of infection of a related virus, either a different serotype of the same virus or of different viruses that show cross-reactivity. ADE is a phenomenon well described in dengue and is considered the main mechanism to trigger the severe form of the disease. Usually, in a secondary DENV infection caused by a different serotype from the primary infection, cross-reactive non-neutralizing antibodies may bind to the heterotypic virus and instead of impairing the virus entry, may help the virus to gain access to the cells through FcγRs, expressed in high levels in cells like monocytes and dendritic cells [48]. As a consequence, virus particles gain facilitated access to permissible cells, while decreasing effector mechanisms like IFNg production by T cells and increasing IL-10 production by macrophages and dendritic cells [48,49], The consequence is an increasing viremia triggering hyperinflammatory responses in the host [50]. Indeed, we could observe that individuals with dengue with warning signs or severe dengue displayed lower activation of T cells [51] associated with increased innate immunity cytokine production, including IFNg production by ILC1 [52]. Although ZIKV-related ADE has been demonstrated to happen in highly controlled experimental settings [53,54,55], its relevance for the clinical presentation of ZIKV infection is still uncertain [56]. On the other hand, previous ZIKV infection was recently demonstrated to be a risk factor for more severe infection caused by dengue serotype 2 in Nicaragua [57] (more details below). Previous ZIKV infection did not appear to affect the severity of the disease caused by other DENV serotypes [57].

On the other hand, T cell-mediated immunity is also shown to play a role in ZIKV infection. T cell activity is key for optimal antibody production [58] and also to eliminating infected cells. CD8+ T cells were found necessary to control ZIKV infection in type I IFN-deficient animals [59,60] and also to protect against CZS in experimental pregnancy models [61]. Although the profile of the immune response associated with virus infections is dominated by IFNγ production, we and others have found mixed profiles associated with ZIKV-infection. For example, IFNγ is found in the peripheral blood of ZIKV-infected patients associated with high levels of IL-10, IL-17A and TNF [62]. Experimental models suggest that an effective immune response against ZIKV is driven by multifunctional CD4+ and CD8+ T cells [59,63]. We have found that ZIKV-specific T cells display a dominant multifunctional profile secreting multiple cytokines, especially IFNg, IL-17A, TNF and IL-10, simultaneously (quadruple-producing T cells) or its combinations [64] (Figure 1). Interestingly, we observed that CD8+ T cells displayed higher frequencies of multifunctional lymphocytes when compared to CD4+ T cells [64]. Indeed, multifunctional T cell responses and cellular immunity have been considered important mechanisms for host resistance against other flaviviruses like DENV [51,65] and immunity to YFV [66,67,68].

Another important factor to consider in immunity against ZIKV and other flavivirus infections is the cross-reactivity of the immune response. Cross-reactivity is well appreciated in DENV infection, since there are four serotypes of DENV (DENV1, DENV2, DENV3 and DENV4) between which are moderate levels of amino acid conservation (around 60–75% at the amino acid level of E protein) [69], making the emergence of broadly neutralizing antibodies and pan-T cell epitopes possible [70,71,72]. This level of genetic proximity causes important cross-reactivity at antibody and T cell levels between different DENV serotypes. The non-neutralizing antibody cross-reactivity between the different serotypes of DENV is considered the main cause of ADE in secondary dengue. Although the levels of similarity between ZIKV and DENV is lower than those observed between DENV serotypes, around 55% of molecular identity at the amino acid level [45], it is still sufficient to cause important cross-reactivity in both, humoral and cellular immunity [45,73,74].

Previous immunity to other flaviviruses can dramatically influence the immune response to ZIKV infection [75,76,77,78]. It has been shown that DENV-experienced individuals displayed consistently higher ZIKV-specific neutralizing antibody titers with low ADE activity compared with DENV-naïve individuals [75]. Interestingly, better antibody protective responses were directly associated with higher levels of ZIKV-specific CD4+ T cells producing IFNγ [75]. Animal data suggest that CD4+ T cells present high levels of cross-reactivity between ZIKV, DENV, WNV and YFV [79]. In addition, CD4+ follicular T cells, which are responsible for the activation of germinal centers and the emergence of high-affinity antibodies, improve ZIKV-specific antibody responses and are important for host resistance during rechallenge [80]. This implies that flavivirus-experienced individuals may display better antibody responses possibly due to faster and improved responses of follicular T cells in providing help to germinal center B cells, when compared to flavivirus-naïve individuals [81].

The clinical implications of such relatedness and cross-reactivity are still the subject of major debate in the literature. For example, it has been suggested that antibodies to any flaviviruses can enhance infection in vitro to almost any other flavivirus at a low enough dilution [82]. Therefore, we can speculate that finding DENV-induced ADE against ZIKV, or vice versa, in vitro, is not surprising because of the cross-reactivity. However, as mentioned, the ADE implication to ZIKV infection is the subject of much debate. Some authors suggest that pre-formed DENV-specific antibodies can cause ADE during a ZIKV infection [53,55,83,84,85], enhancing vertical transmission and triggering microcephaly in pups during pregnancy [86]. In contrast, data show that neutralizing anti-DENV cross-reactive antibodies can also neutralize ZIKV [87,88] and some data suggest a relationship between anti-DENV antibodies and protection from ZIKV infection in humans [89]. Meanwhile, some have suggested that a previous DENV infection in mice can prevent a lethal ZIKV challenge, not because of antibodies, but due to CD8+ T cells [90]. Interestingly, an epidemiological study in Brazil found that ZIKV-related microcephaly was more common in Brazilian regions with the least coverage of YFV vaccination [91] suggesting a role for cross-reactivity between YFV immunity. In agreement, Vicente et al. demonstrated that immunization against YFV using 17DD vaccine can decrease cerebral virus load, prevent neurological manifestations, weight loss and mortality in a fatal murine model of ZIKV infection by mechanisms possibly associated with cross-reactive cell-mediated immunity [92]. Indeed, YFV vaccination leads to the emergence of ZIKV-specific CD8 T cells [93]. Finally, other authors sustain that pre-formed immunity against DENV or YFV will not affect a subsequent ZIKV infection as found in non-human primates [94,95] or in human subjects [96]. On the other hand, there are data emerging showing that a primary ZIKV exposure may have a larger impact on subsequent DENV infection due to ADE [57].

## 4. Implications of Flaviviruses Immunity on ZIKV Vaccine Development

The interaction of ZIKV immunity with other flaviviruses, and vice versa, may have important implications for vaccine development. The challenges for DENV vaccine development are examples of how difficult it might be to develop a vaccine for ZIKV. A vaccine for DENV must concomitantly induce homotypic neutralizing antibodies to each of the serotypes. If not, partial immunity against one of the serotypes with the presence of non-neutralizing antibodies may increase the risk of ADE and severe dengue. This happened to CYD (Denvaxia©), the dengue vaccine of Sanofi Pasteur, which induced antibodies that were not balanced between the four serotypes in individuals who were not previously exposed to natural DENV infection [97].

CYD tetravalent vaccine is a vaccine combination of four recombinant viral vector vaccines using yellow fever 17DD structure to express the structural proteins (prM and E proteins) of each DENV serotype [98]. This vaccine was able to induce neutralizing antibodies against the four DENV serotypes in more than 90% of vaccinees [99]; however, homotypic neutralizing antibody against DENV-4 component of the vaccine was dominant [97]. The DENV-1 and DENV-2 components were poorly infectious and the majority of DENV-1 and DENV-2 antibodies induced by the vaccine were heterotypic [97,100]. The vaccine induced some levels of IFNγ-producing DENV-specific CD4+ T cells directed to DENV E protein [98] and possibly to boost, by cross-reactivity between YFV and DENV antigens, some preexisting levels of DENV NS3 T cells [101]. Despite the capacity to induce neutralizing antibodies and IFNγ against DENV [102,103], CYD did not perform well in in phase III clinical efficacy trials. CYD displayed a general efficacy of 50%, 35–42%, 74–78% and 75–77% against DENV 1, 2, 3 and 4, respectively [99,104,105]. Its protection was better in the dengue-experienced population, in which it reached 70% efficacy, but only 35% in dengue-naïve individuals [104]. Unfortunately, an excess number of dengue hospitalizations occurred in year 3 of the trial in those volunteers who received the CYD vaccine compared to those who received a placebo in the first year of the trial. This was observed more frequently in 9-year-old children and below [106]. Detailed analysis of CYD immunogenicity data demonstrated that: (1) homotypic anti-DENV4 antibodies were dominant, while the other serotypes were neutralized mostly by cross-reactive heterotypic antibodies [97]; (2) individuals with higher titers of neutralizing antibodies were more protected than individuals with lower titers [107]; and (3) most of the CD4+ and CD8+ T cells induced by the vaccine were directed against the yellow fever component [107,108,109]. Some important conclusions can be drawn from these data: (a) the mere presence of neutralizing antibodies is not a sufficient correlate of protection for DENV, and perhaps for other flaviviruses like ZIKV, as unbalanced homotypic versus heterotypic antibody titers lead to higher titers of heterotypic, non-neutralizing, potentially enhancing antibodies; and (b) T cell mediated immunity seems to play an important role as a mechanism of protection, since this was a major missing mechanism of CYD.

**Figure 2:**
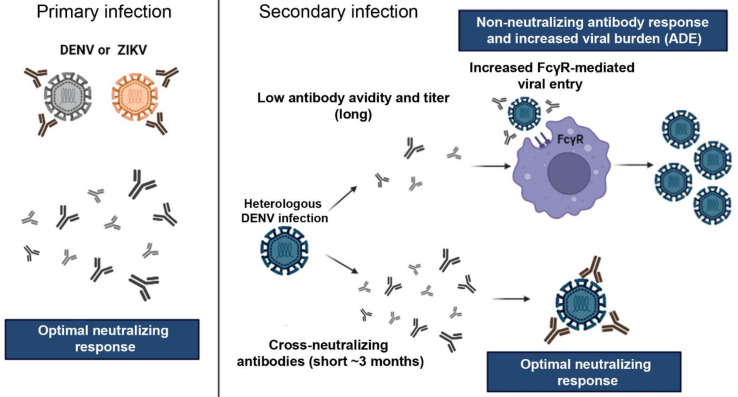
Antibody response and cross-reaction effect between DENV and ZIKV. Primary infection by one of the viruses can promote the production of long-lasting neutralizing antibodies the homologous virus and short-lived cross-reactive neutralizing antibodies, i.e., in the first few months (usually 3 months) after infection, neutralizing antibodies also neutralizes heterologous related viruses. However, after 3 few months, cross-neutralization is lost and cross-reactive non-neutralizing antibodies may enhance the infection during a secondary exposure by a heterologous related virus, for example, a second distinct DENV serotype, or a DENV infection following a primary ZIKV infection. Virus particles opsonized with non-neutralizing antibodies have facilitated access to permissive cells via FcγR which causes enhanced virus proliferation and increased viral load. This phenomenon is known as antibody-dependent enhancement (ADE). On the other hand, heterologous infections after short periods between the primary and secondary infections can induce virus neutralization by cross-reactive antibodies. Abbreviations: ADE—antibody-dependent enhancement. Images from Servier Medical Art, licensed under a Creative Common Attribution 3.0 Generic License (http://smart.servier.com/; accessed on 10 Februrary 2022).

Considering immunization against ZIKV, the scenario is not less complex. If the implications of DENV-induced ZIKV ADE are uncertain, there is evidence to suggest that cross-reactivity of anti-ZIKV antibodies may cause ADE against DENV, and this could be a problem in vaccination strategies (Figure 2). It has been demonstrated that ZIKV immunity can precipitate ADE against DENV in vitro [110,111] and in the pediatric population [57]. Katzelnick et al. observed in a careful Nicaraguan pediatric cohort that previous exposure to DENV or ZIKV infection in naïve children, or one ZIKV infection in one DENV infection-experience children, increased the risk of severe disease in a subsequent DENV2 infection. Their findings also found that while high titers of anti-DENV antibodies (developed after multiple DENV infections) have protective effects, intermediate anti-DENV titers precipitate ADE in subsequent DENV orZIKV infections [57]. The implication of this finding for vaccine development is clear: immunization against DENV must be addressed in the context of ZIKV immunization. DENV/ZIKV immunization can be achieved by sequential immunization, i.e., vaccination against DENV and then against ZIKV [112], or preferentially by the combination of dengue and Zika vaccines.

A major rationale for the combination of vaccines, when possible, is the simplification of the immunization schedule [113]. The number of shots necessary to cover immunization programs in developed or even Low–Middle Income Countries (LMIC) can decrease compliance and increase costs. On the other hand, the combination of vaccines has been a mechanism to reach adequate levels of immunization to as many as 14 diseases in infants below two years old. For instance, the combination of vaccines into a single shot such as pentavalent DtaP-HepB-IPV and trivalent MMR has contributed significantly to simplification of immunization schedules, increased compliance and cost reduction because of delivery efforts and vaccination campaigns are optimized to many vaccines at once [113].

DENV and ZIKV often co-circulate in endemic countries because the mosquito vector is the same, *A. Aegypti* or *A. albopictus* [2], and the most affected populations live in developing countries where financial resources for public health are limited. There are strong epidemiological, compliance and cost-bases for the combination of DENV and ZIKV vaccines into one single shot, especially targeting the possibility of a robust immune response to such a combination due to cross-reactivity between ZIKV and DENV. If the immunization against ZIKV poses a threat for severe dengue because of cross-reactive non-neutralizing antibodies, the combination of ZIKV and DENV vaccines may provide further protection against both diseases. Again, a great lesson comes from dengue. The live attenuated dengue vaccine (LADV) TV003 induces antibodies that are mostly homotypic, with little cross-reactivity, for each of the four DENV serotypes [114] and induces T cell responses to highly conserved CD8 epitopes [115]. The addition of a ZIKV vaccine in the mix, or a concomitant immunization, has the potential to induce ZIKV-specific antibodies and to also add ZIKV T cell epitopes to the mix, further selecting highly conserved epitopes and improving both DENV and ZIKV immune responses.

## 5. Current Challenges and Solutions: A Role for Controlled Human Infection Model of ZIKV

**Figure 3:**
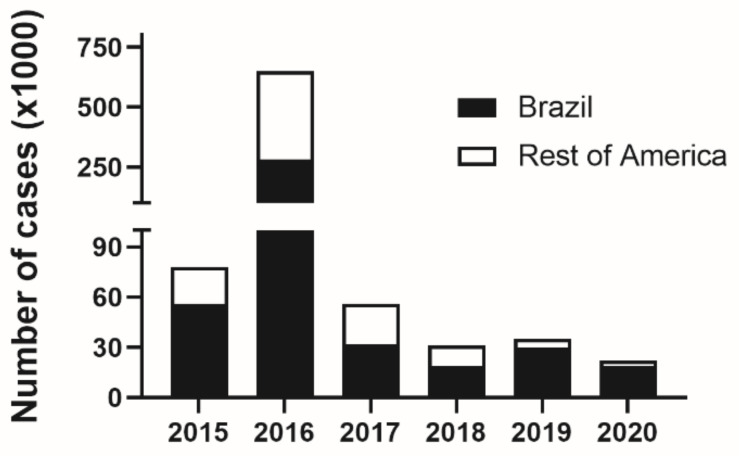
Number of zika case notifications in Latin America and Brazil in the years between 2015 and 2020. Number of ZIKV infections were accessed in PAHO website (https://www3.paho.org/data/index.php/es/temas/indicadores-zika.html; accessed on 20 December 2021) and Brazilian DataSUS (https://datasus.saude.gov.br/informacoes-de-saude-tabnet/; accessed on 20 December 2021).

The need for a Zika vaccine is still a public health necessity. Although ZIKV circulation has shown a dramatic drop since 2016 (Figure 3), the numbers of pregnancy complications associated with Zika are still a concern [22,116,117] and a vaccine aiming to protect pregnancies in Zika endemic countries is of great interest. Several vaccines are under development, mostly in the preclinical phase (88 vaccines), a fair number in phase I (20 vaccines) and only one vaccine in phase II clinical trials [118]. Although there is a reasonable pipeline of ZIKV vaccines, the slow pace of development and the low numbers of ZIKV infections might make it difficult to perform a traditional Phase III efficacy trial.

After the peak of the Zika outbreak in 2016, the commercial interest in a Zika vaccine considerably dimmed. Despite its relevance for public health, especially in women of child-bearing age, lower investment decreased the pace of a Zika vaccine development. In addition, Brazil reports only between 15,000 and 31,000 Zika cases per year since 2018. The estimated attack rate for the population of Brazil is too low for a Phase III vaccine efficacy trial and the probability of individuals participating in a ZIKV vaccine trial being actually exposed to ZIKV is too low [119].

Controlled human infection “models” (CHIM) are great tools for vaccine development. CHIM refers to the use of iatrogenic infection of humans for study purposes. Models of controlled infection are usually developed to study the clinical aspects and physiopathology of infection and to develop drugs or vaccines for specific pathogens [120]. For instance, variolation was a “primitive” procedure of controlled human infection to induce active immunization of a subject against the natural infection with variola. On the other hand, the same procedure was used by Jenner as a model to test the efficacy of immunization with bovine variola, therefore, creating the first use of CHIM which gave the world the first vaccine.

Estimates account that more than 20,000 volunteers have participated in CHIM studies since World War II and that these studies have helped to characterize aspects of the clinical presentation and evolution, immune responses, microbiology and pathogenesis of many infections. Furthermore, CHIM has contributed to the development of a number of vaccines, making a tremendous impact on public health. Vaccines that have utilized CHIMs include those for influenza, shigella, enteric fever, malaria, campylobacter, cholera and RSV, among others [121].

The use of CHIM can benefit vaccine development as they can down-select candidates so that only the most promising candidates are further evaluated in Phase II and III clinical trials. While formal efficacy trials (Phase III) need to recruit tens of thousands of individuals and follow them until the efficacy endpoint is met, which may vary according to the incidence of the infection, CHIMs are designed to ensure a high attack rate allowing for a rapid assessment of efficacy. For example, malaria-controlled infection models are now a standard step in malaria vaccine development to assess if a vaccine should advance to larger Phase II and Phase III clinical trials. The malaria-controlled human infection model was utilized to select the antigen RTS,S (a *Plasmodium falciparum* circumsporozoite protein (S) fused with hepatitis B antigen (RTS)) for the Mosquirix vaccine [122,123]. The efficacy endpoint for the malaria CHIM is the prevention of infection. A malaria CHIM may recruit 10–20 volunteers in each group (vaccinated and placebo), proceed with vaccination and infect all the groups. Volunteers are evaluated daily (or more frequently) for parasitemia (i.e., the appearance of the sporozoites in the blood). Once parasitemia occurs, the volunteer is treated with anti-malaria medication, even before they become symptomatic, making the procedure highly informative for science and safe for the participants. If the vaccine were to work, the placebo group should become positive while the vaccinated group would be negative at the same timepoint. It is important to note that even if a vaccine demonstrates efficacy in a CHIM study, it still must be evaluated in thousands of volunteers to ensure safety.

The use of CHIM for vaccine development, although very common in developed countries like USA and England, faces many barriers in developing countries. For example, Vietnam has laws that forbid the intentional infection of a subject, as stated in constitutional amendment No. 51/2001/QH10, Article 8.1. Other developing countries may see the use of CHIM as detrimental to their own dignity, or still other countries may even face the lack of regulatory expertise which would enable CHIMs to be performed ethically. Nevertheless, the use of CHIM can bring many benefits to developing countries as a tool that can speed up the development of vaccines and therapeutics for public health problems important to them, including Zika.

So far, only one vaccine, Vaxchora, has been approved by the FDA based on CHIM efficacy data. Vaxchora is a cholera vaccine that was approved for travelers going to cholera endemic areas. Since it demonstrated good efficacy against cholera challenge in naïve non-endemic individuals [124,125,126,127], the FDA has approved its use by travelers, despite poor performance in endemic settings [128]. The case of Vaxchora also illustrates the importance of performing accelerated efficacy studies, using CHIM, in endemic settings since genetic and exposition history may change the immunogenicity to the vaccine and host response to the pathogen.

The U.S. National Institutes of Health are developing a CHIM for Zika to aid in vaccine development and evaluation. The proposal for a Zika CHIM underwent ethical review in late 2016. The committee determined that a Zika CHIM was not needed at that time for vaccine development as the Zika outbreak was causing large numbers of cases in Latin America. However, after Zika circulation essentially came to a halt in late 2017, the need for a Zika CHIM was reconsidered and determined to be a useful tool for vaccine development [129]. A Zika CHIM study is underway and will evaluate two strains of ZIKV recovered from persons who developed uncomplicated ZIKV infection (clinicaltrials.gov: NCT05123222). The study population will be composed of healthy non-pregnant, non-breastfeeding women (18–40 years old), who will be housed in an inpatient unit during the viremic period. The study will evaluate up to three different doses of the ZIKV and follow the study participants for up to 6 months following inoculation for assessment of safety (including GBS) and immunogenicity. During this period, participants must agree to use acceptable effective birth control measures. The aim of the study is to identify a suitable challenge virus to evaluate vaccine efficacy early in development. This ZIKV CHIM will also be used to evaluate the protective efficacy of the tetravalent DENV vaccine TV003 against subsequent ZIKV infection as well as evaluating the effects of ZIKV antibody on subsequent DENV infection and vice versa.

## 6. Conclusions

ZIKV caused a major epidemic in 2015 in Latin America, but its incidence decreased and has stabilized to around 15,000–30,000 infections yearly. Those numbers are likely underestimated since around 5–10% of microcephaly cases in Brazil seem to be associated with CZS [22,116,117]. Immunological data suggest broad cross-reactive immunity of ZIKV with other flaviviruses, especially DENV. This may have important implications for flavivirus immunization programs. The development of a ZIKV vaccine should consider the immunological cross-reactivity between ZIKV and other flaviviruses, especially the possibility of enhancing the severity of subsequent DENV infection. Additionally, the currently low yearly ZIKV incidence will make it difficult to conduct formal Phase III clinical trials. For these reasons, there is a strong rationale for developing a combined DENV and ZIKV vaccine and using the ZIKV CHIM to assess the efficacy of a Zika vaccine. The development and use of a Zika CHIM to evaluate the protective efficacy of potential ZIKV vaccines is of great interest to countries where Zika is endemic and is still causing CZS, like Brazil.

## Data Availability

Not applicable.
